# A case of silicone oil adhered to the retinal surface via perfluorocarbon liquid

**DOI:** 10.1186/s12886-018-0745-y

**Published:** 2018-03-27

**Authors:** Masanori Fukumoto, Yui Nishida, Teruyo Kida, Takaki Sato, Takatoshi Kobayashi, Tsunehiko Ikeda

**Affiliations:** Department of Ophthalmology, Osaka Medical College, 2-7 Daigaku-machi, Takatsuki-City, Osaka 569-8686 Japan

**Keywords:** Silicone oil (SO), Perfluorocarbon liquid (PFCL), Vitrectomy, Rhegmatogenous retinal detachment

## Abstract

**Background:**

Perfluorocarbon liquid (PFCL) is widely used as an intraoperative heavy tamponade to flatten the retina and is replaced with silicone oil (SO) at the end of the surgery. Due to the long tamponade period, the SO is known to remain attached to the retina at the time of removal, and is commonly termed “sticky oil”. The aim of this present study was to report a case of SO stickily attached to the retina via PFCL without tamponade period.

**Case presentation:**

A 39-year-old male was referred to our hospital due to decreased vision and visual field defect in his right eye. Upon examination, he was diagnosed with rhegmatogenous retinal detachment in that eye. For treatment, he underwent vitrectomy with the use of PFCL and SO. The direct exchange of PFCL with SO resulted in residual subretinal fluid, so we subsequently attempted to remove the SO. However, a SO bubble adhering to the PFCL was visible on the posterior pole. After aspiration of the PFCL beneath the sticky SO, the SO was easily separated and removed from the retina.

**Conclusions:**

Our findings show that SO can become tightly adhered to the retinal surface via PFCL during vitrectomy, and that the sticky SO can be safely removed via aspiration of the PFCL layer underneath the SO.

## Background

Over the past few decades, silicone oil (SO) has been widely used in vitreoretinal surgery as a long-term tamponade in the surgical treatment of patients with retinal detachment [1]. Perfluorocarbon liquid (PFCL) has also been used to flatten the detached retina during the surgery [2, 3]. Due to its retinal toxicity, PFCL should be completely removed during surgery. The removal of PFCL is frequently performed by a direct exchange with SO, especially in severe cases such as a giant retinal break [4]. However, the PFCL can come into direct contact with the SO during the exchange. Although there is usually no negative interaction between/in these two materials when attached, it has recently been reported that PFCL can form a viscous substance with SO after a long-term period of surgical tamponade [5].

In this study, we present a case in which SO became adhered to the surface of the retinal via PFCL during vitrectomy.

## Case presentation

**Fig. 1:**
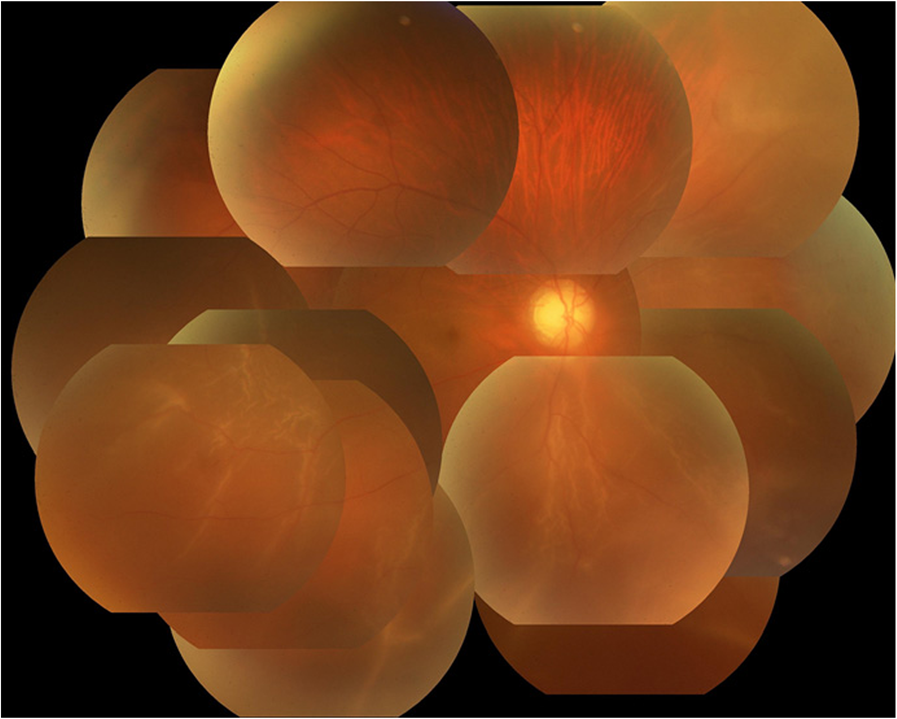
A fundus color photograph of the right eye of the 39-year-old male patient obtained at the first examination. Bullous rhegmatogenous retinal detachment in the inferior, nasal, and temporal quadrants was observed

**Fig. 2:**
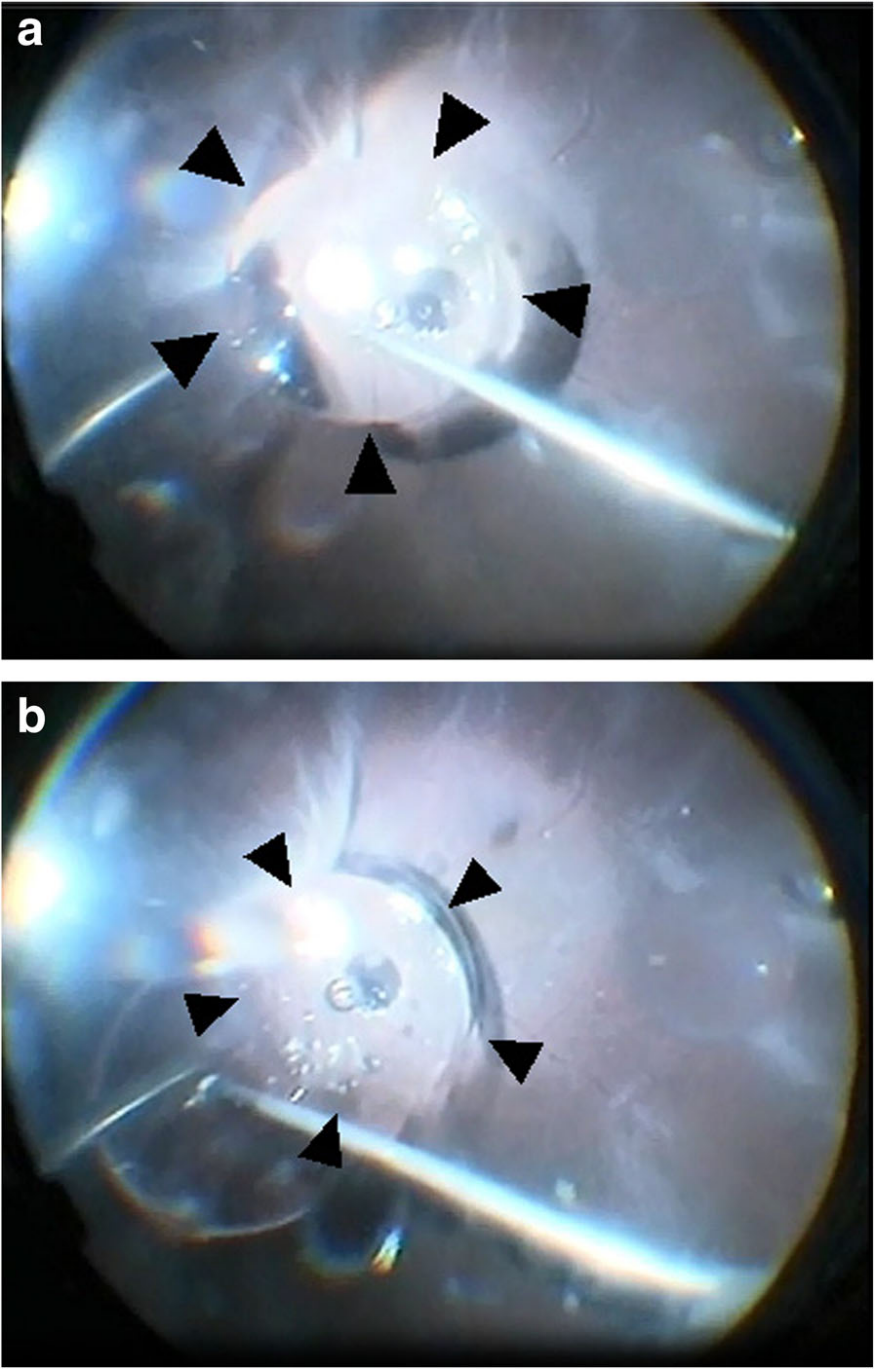
Intraoperative findings. **a** A silicon oil (SO) bubble adhered to the posterior pole of the retina of the patient’s right eye. The SO could not be removed by aspiration via the use of a backflush needle (*). Arrowheads indicate the base of the SO bubble. **b** The SO followed the movement of the backflush needle by continuous active suction. The base of the SO (arrowheads) was stable

A 39-year-old male was referred to our hospital due to decreased vision and visual field defect in his right eye. Upon examination, this right-eye visual acuity was 0.03 and bullous rhegmatogenous retinal detachment was found in three quadrants, yet not in the upper part of the retina (Fig. 1). For treatment, we performed the standard 4-port pars plana vitrectomy using the 25-gauge system. Following the core vitrectomy and artificial posterior vitreous detachment, we injected PFCL to flatten the detached retina. After endophotocoagulation was performed around the retinal break, we directly replaced the PFCL with SO. Due to misdirection of aqueous fluid into the subretinal space during the replacement, we attempted to remove the SO and exchange it with aqueous solution. However, a small amount of SO was found to be adhered to the posterior pole of the retinal surface, and it was impossible to remove the bubble of SO by active suction at the vitreous center (Fig. 2). Although the shape of the SO bubble was easily deformed, the location of the sticky SO remained unchanged.

The PFCL layer between the SO and the underlying retina was easily aspirated and the SO was released from the retinal surface. Post release, the SO was relocated to the anterior chamber and safely removed through a corneal limbal incision. After complete removal of the SO, pneumatic retinal replacement and laser photocoagulation were performed, and sulfur hexafluoride gas was used as a temporary intraocular tamponade. At 7-months postoperative, the patient’s retina remained attached and his corrected visual acuity improved to 0.7.

## In vitro experiment

**Fig. 3:**
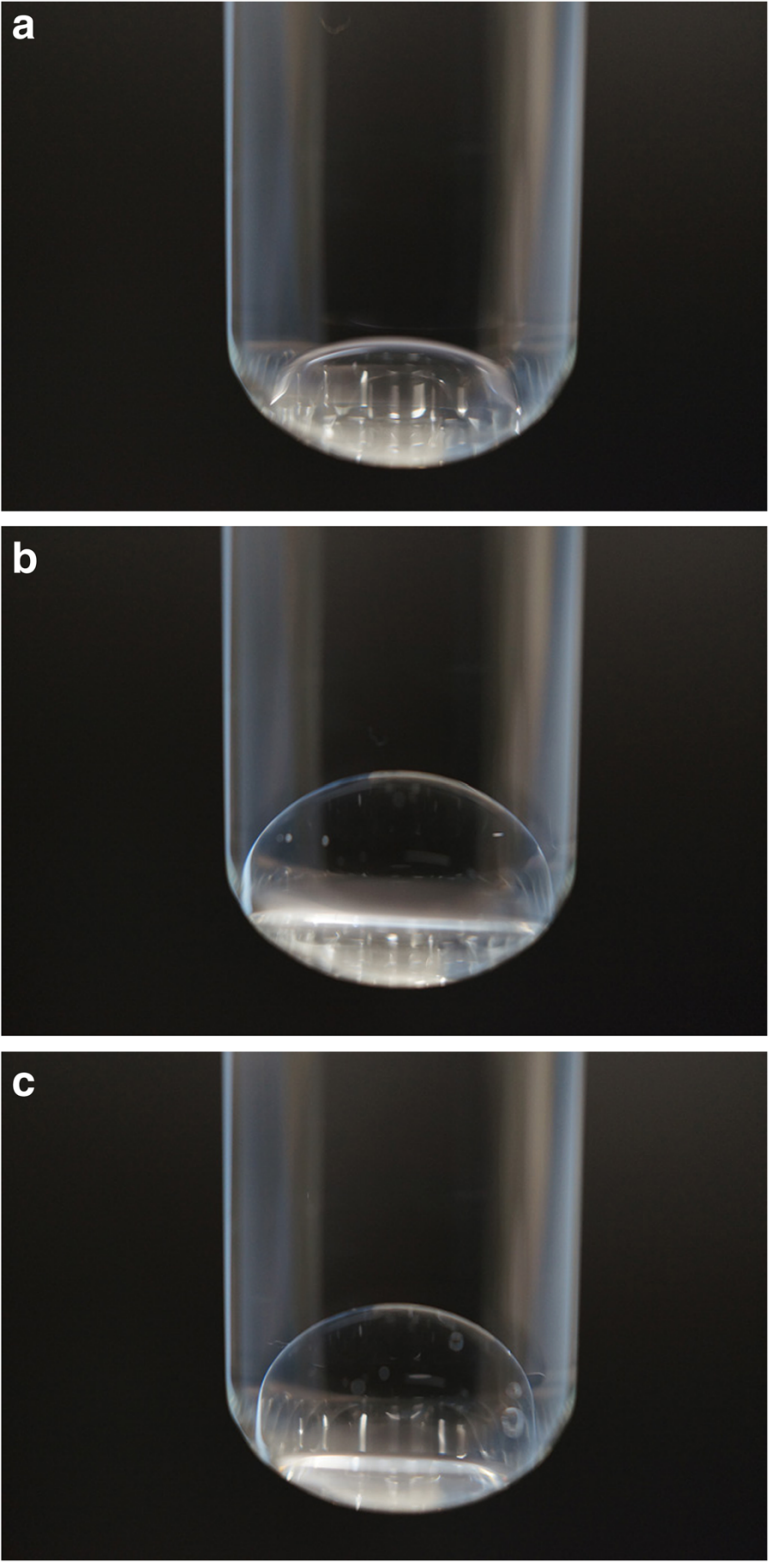
In vitro experiment. **a** A perfluorocarbon liquid (PFCL) bubble in a glass test tube filled with balanced salt solution (BSS). PFCL (*) was placed at the bottom of the glass test tube filled with BSS (†). **b** Interaction of the SO and PFCL bubbles. SO (††) covered the surface of the PFCL (*) and sunk in the BSS (†). **c** The SO (††) changed shape into a spherical form after partial removal of the PFCL (*) in the BSS (†)

We performed an in vitro experiment to investigate the interaction between PFCL and SO. For that experiment, a glass test-tube was filled with balanced salt solution (BSS). Next, 0.2 ml of PFCL was introduced into the solution and placed onto the bottom of the tube (Fig. 3a). Next, 0.3 ml of SO was transferred into the tube and came into contact with the PFCL, and was observed to spread and cover the surface of the PFCL (Fig. 3b). After partial removal of the PFCL, the attached surface of the two substances decreased, and the SO changed shape into a spherical form (Fig. 3c).

## Discussion

In this study, we report a case in which an SO bubble became tightly adhered to the retinal surface during vitrectomy. To the best of our knowledge, this is the first report of sticky SO becoming tightly adhered to the retinal surface via PFCL. The difficulty of removing SO at the end of the tamponade period has previously been reported. In a retrospective study, Veckeneer et al. reported 28 of 234 cases (12.0%) in which patches of sticky SO remnants adhered to the retina within the posterior pole during removal of the SO [5]. Sleep et al. encountered a case in which the SO and PFCL became mixed and was difficult to remove [6]. In all of the previously reported cases, the findings similarly showed that the SO became sticky after long-term tamponade periods. In this present case, and in contrast to the previous reports, the SO became sticky during its removal immediately after the direct exchange of PFCL with SO.

It should be noted that the water layer between PFCL and SO can flow into the subretinal space through the retina tear during the direct exchange of PFCL with SO. To avoid such a phenomenon, it is recommended that the aqueous interface be removed in the early stage of the exchange. Complete removal of aqueous liquid can result in a direct contact between PFCL and SO. Subsequently, these two substances can become adhered to each other due to the nonallele nature of the two phases [7]. In this present case, subretinal fluid was observed during the exchange period. We theorized that misdirection of the water layer into the subretinal space might have contributed to the complete attachment of PFCL and SO.

In our in vitro experiment, the three components (i.e., SO, PFCL, and aqueous liquid) were found to form their shapes to reach the point where their total energy would be lowest, and we found that the SO fully covered the surface of PFCL. To separate SO from PFCL, the applied energy must exceed the adhesion force between the SO and the PFCL. Dresp et al. and Ghoraba et al. reported that the cohesion of SO is lower than the adhesion forces, and concluded that aspiration of SO from the vitreous cavity after the direct exchange of PFCL with SO can be extremely difficult [8, 9].

Microincision vitreous surgery is gaining popularity, and is being applied to complicated cases more than ever before. Due to the small diameter of available cannula devices, high aspiration forces are necessary to remove high-viscosity materials such as SO. To remove sticky SO droplets, the tip of the cannula should be placed close to the retinal surface. It is known that strong suction forces adjacent to the retina can lead to the development of an iatrogenic retinal tear. In contrast to SO, PFCL can be removed via the use of a relatively small suction force. In our case, SO was easily removed from the areas of the retina that were coated with PFCL by aspirating the PFCL layer beneath the SO.

## Conclusion

Our findings show that in cases involving a tight adhesion of SO on the surface of the retina during microincision vitreous surgery, the SO bubble can be easily removed by aspiration of the PFCL layer underneath the SO.

## Abbreviations

BSS: Balanced salt solution
PFCL: Perfluorocarbon liquid
SO: Silicone oil
